# Kinesin Eg5 Selective Inhibition by Newly Synthesized Molecules as an Alternative Approach to Counteract Breast Cancer Progression: An In Vitro Study

**DOI:** 10.3390/biology11101450

**Published:** 2022-10-02

**Authors:** Alessia Ricci, Amelia Cataldi, Simone Carradori, Susi Zara

**Affiliations:** Department of Pharmacy, “G. d’Annunzio” University of Chieti−Pescara, Via dei Vestini 31, 66100 Chieti, Italy

**Keywords:** breast adenocarcinoma, kinesin Eg5 inhibitors, angiogenesis, apoptosis, cell invasion, cell migration, K858

## Abstract

**Simple Summary:**

Breast cancer is the most widespread and diagnosed cancer in women. This study tested newly synthesized Eg5 inhibitors to find valid therapeutic alternatives to common drugs able to counteract breast cancer progression. Our results evidence that thiadiazole-based Eg5 inhibitors, **2** and **41**, appear to negatively modulate proliferation, invasiveness and migration of cancer cells, affecting also the angiogenesis process and the remodeling of the extracellular matrix, thus representing a new promising drug strategy to control breast cancer.

**Abstract:**

Breast cancer (BC) is one of the most diagnosed cancers in women. Recently, a promising target for BC treatment was found in kinesin Eg5, a mitotic motor protein that allows bipolar spindle formation and cell replication. Thus, the aim of this work was to evaluate the effects of novel thiadiazoline-based Eg5 inhibitors, analogs of K858, in an in vitro model of BC (MCF7 cell line). Compounds **2** and **41** were selected for their better profile as they reduce MCF7 viability at lower concentrations and with minimal effect on non-tumoral cells with respect to K858. Compounds **2** and **41** counteract MCF7 migration by negatively modulating the NF-kB/MMP-9 pathway. The expression of HIF-1α and VEGF appeared also reduced by **2** and **41** administration, thus preventing the recruitment of the molecular cascade involved in angiogenesis promotion. In addition, **2** provokes an increased caspase-3 activation thus triggering the MCF7 apoptotic event, while **41** and K858 seem to induce the necrosis axis, as disclosed by the increased expression of PARP. These results allow us to argue that **2** and **41** are able to simultaneously intervene on pivotal molecular signaling involved in breast cancer progression, leading to the assumption that Eg5 inhibition can represent a valid approach to counteract BC progression.

## 1. Introduction

Breast cancer (BC) is one of the most widespread cancers among women [1] and in 2020, 2.3 million new cases were recorded (11.7% of incidence and 6.9% of mortality), surpassing lung cancer (11.4% of incidence) as the main cause of cancer death worldwide [2]. This type of tumor shows a high grade of variability and based on molecular and histological characteristics, different types of BC can be identified: BC positive for both estrogen and progesterone receptors (ER^+^, PR^+^), named Luminal A, BC positive for ER, PR and for human epidermal receptor 2 (HER2^+^) (named Luminal B), BC positive only for HER2 (named HER2-overexpression) and triple-negative BC (TNBC, negative for ER, PR, and HER2 receptors), also called Basal-like [3,4]. There are different approaches to treat patients with BC such as surgical resection of the tumor and regional lymph node (axillary lymph node) and postoperative radiation for patients with non-metastatic BC, systematic therapy specific for histological BC characteristics (endocrine therapy, chemotherapy, immunotherapy) [5], and combination therapy [6].

Kinesin proteins superfamily (KIFs) is the first large protein superfamily in mammals, consisting of 14 different subfamilies, with different motor roles in the cells. They allow various cellular functions due to their movement along the microtubules provided by their motor domain, in an adenosine triphosphate (ATP)-dependent manner [7]. Miki et al. defined KIFs as the “hub” proteins in the intracellular transport system as they are essential for intracellular transportation. In addition to this pivotal function, there are specific categories of kinesins, called mitotic kinesins, which provide chromosomal and spindle movements during key biological processes such as mitosis and meiosis [8,9]. Among them, a relevant role is played by kinesin Eg5, also known as kinesin spindle protein (KSP). It is a microtubule plus-end directed protein positioned between two polar microtubules, which allows their overlapping at the equator of the cell and the separation of the two centrosomes at the opposite poles of the cell, with a bipolar spindle formation during cell division [10]. Because of this essential activity during cell replication and for its overexpression in different types of tumors [11] such as gallbladder cancer [12], glioma [13], lung cancer [14], neuroblastoma [15], and others, it became a promising target for cancer therapy [16]. A high expression rate of Eg5 was also measured in breast cancer leading to assume that this kinesin can represent an oncogene in BC and a new potential prognostic marker [17,18]. Based on this knowledge, different Eg5 inhibitors were tested on BC as a new possible therapeutic strategy [19,20] finding a correlation between Eg5 activity and ER^+^/PR^+^ BC occurrence, thus indicating Eg5 inhibitor therapy as a promising strategy to treat patients with ER^+^/PR^+^ BC rather than hormonal therapies [21,22]. Eg5 inhibitors were demonstrated to act by blocking centrosome separation, activating the spindle checkpoint, and inducing cellular mitotic arrest. This antiproliferative effect was less evident in non-transformed cells, where Eg5 inhibitors did not furnish mitotic cell death, polyploidization followed by senescence, or formation of micronuclei. In addition, they were characterized by minimal abnormalities in chromosomes and the absence of neurotoxicity in cell-based and in vivo assays with respect to other tubulin-interacting agents such as taxanes or vinca alkaloids. The final effect of Eg5 inhibitors was the accumulation of mitotic cells with monopolar spindles also in xenografts models of cancer. Among these compounds, K858 is a racemic ATP-uncompetitive inhibitor of kinesin Eg5 (IC_50_ = 1.3 μM), which can interact at an allosteric inhibitor-binding pocket composed of helix α2, loop L5 and helix α3. Starting from this lead compound and after the assessment of the structure determination of two crystal forms of the ternary Eg5-ADP-K858 complex, we chemically obtained a large library of derivatives keeping constant the 1,3,4-thiadiazole scaffold [23]. In our previous works, seven of the most potent thiadiazoline-based Eg5 inhibitors, analogs of K858, have been already tested in an in vitro model of stomach tumor, identifying two of them, namely **2** and **41**, with a better profile in terms of cancer cells proliferation control, reduction of cell invasiveness and promotion of the apoptotic event [24,25].

The aim of this work is to evaluate the effect of thiadiazoline Eg5 inhibitors in an in vitro tumoral model of an ER^+^/PR^+^ BC cell line (MCF7), based on the correlation existing between Eg5 and ER^+^ BC. Indeed, the authors demonstrated that downregulation of ER by the administration of fulvestrant in two different ER^+^ BC cell lines (MCF7 and ZR-75) induced downregulation of Eg5, while its administration had no impact on Eg5 expression in an ER^−^ breast cancer cell line (MDA-231). Additionally, the administration of estrogen in MCF7 cell lines treated with fulvestrant-induced Eg5 expression has been also evidenced. In particular, we focused on the effect of newly synthesized Eg5 inhibitors on proliferation, invasion, migration processes, and on apoptosis–necrosis occurrence.

## 2. Materials and Methods

### 2.1. Chemistry

Newly thiadiazoline Eg5 inhibitors, analogs of their parent compound K858, were synthesized, purified (≥96% purity), and characterized, as already reported [26]. The compounds were kept as a 50 mM stock solution in dimethyl sulfoxide (DMSO) at −20 °C.

### 2.2. Cell Cultures

Human breast cancer cells MCF7 (HTB-22™) were purchased from ATCC (ATCC, Rockville, MD, USA) and cultured in Dulbecco’s modified Eagle’s medium (DMEM) high glucose supplemented with 10% of fetal bovine serum (FBS) and 1% of penicillin/streptomycin (all purchased from EuroClone, Milan, Italy). MCF7 were kept in a humidified incubator at 37 °C with 5% CO_2_.

Human gingival fibroblasts (HGFs) were obtained from 10 donors which were not affected by periodontal and systemic diseases. Before undergoing third molar surgical extraction, donors signed the informed consent in accordance with the Italian Legislation and with Ethical Principles for Medical Research including the Human Subjects code of the World Medical Association (Declaration of Helsinki). The project received the Local Ethical Committee of the University of Chieti approval on 31 March 2016 (Chieti, Italy; protocol number 1173). After withdrawal, gingiva samples were placed in DMEM, rinsed in phosphate saline buffer (PBS), cut into smaller pieces, and cultured in DMEM, with 10% FBS, 1% penicillin/streptomycin, and 1% fungizone (all purchased from EuroClone, Milan, Italy). Cells were maintained in a humidified incubator at 37 °C with 5% CO_2_. After 10 days fungizone was removed from culture medium and cells were used for biological experiments (passage 7–14), as elsewhere reported [27].

### 2.3. MTT Assay

MCF7 and HGF metabolic activity were measured by the MTT (3-(4,5-dimethylthiazol-2-yl)-2,5-diphenyltetrazolium bromide) test (Merck Life Science, Milan Italy). MCF7 cells were seeded in a 96-well cell culture plate at density of 10,000 /well. For the initial screening, the MCF7 metabolic activity was evaluated after 24, 48, and 72 h of treatment with newly synthesized Eg5 inhibitors **2**, **4**, **26**, **30**, **31**, **41,** and **44** at 0.5, 5, 50, 100 µM and with the parent compound K858 at 0.5, 5 and 50 µM. Then, after having selected compounds **2**, **30,** and **41** for their better profile, a further MTT test was performed, at 1.560, 3.125, 6.25, 12.5, 25, 50, and 100 µM, after 48 and 72 h of treatment.

HGFs were seeded in a 96-well cell culture plate at a density of 7000 /well; their metabolic activity was evaluated after 24 and 48 h of treatment with **2** at 1.5 µM, **41** at 5 µM, and K858 at 10 µM. At the established experimental times, the culture medium was replaced by a new one added with 0.5 mg/mL MTT and incubated in the presence of cells at 37 °C for 5 h. The plate was then probed with DMSO for 30 min to allow violet formazan salt dissolution, formed after the reduction of MTT by viable cells. The colored solution was read at 540 nm by a GO microplate spectrophotometer (Thermo Fisher Scientific, Waltham, MA, USA). Metabolically active cell percentage was obtained by normalizing obtained values with values of DMSO-treated cells.

### 2.4. Cytotoxicity Assay (LDH Assay)

To estimate membrane integrity and to quantify MCF7 cytotoxicity occurrence after treatment with **2** 1.5 µM, **41** 5 µM, and K858 10 µM, lactate dehydrogenase (LDH) leakage within the medium was measured by means of “CytoTox 96 non-radioactive cytotoxicity assay” (Promega, Madison, WI, USA), following the manufacturer’s instructions. The assay was carried out after 24 and 48 h of treatment and the measured LDH leakage in each well was normalized with the optical density values obtained from MTT test.

### 2.5. Transwell Invasion and Migration Assay

Both MCF7 cell invasion and migration assays were carried out by using a 24-well Transwell Boyden chamber consisting of inserts with 8 µm pore size membranes (Corning, Lowell, MA, USA).

For MCF7 cell invasion assay, a thin layer of ECM Gel (Merck Life Science, Milan, Italy), with a final concentration of 0.4 mg/mL (stock solution: 8–12 mg/mL), was used to coat the pores of membranes insert, mimicking the extracellular matrix. An amount of 150 µL of 0.4 mg/mL of ECM gel diluted in ice-cold serum-free DMEM was placed in the upper chamber compartment of the insert and incubated for 2 h at 37 °C to allow gel to solidify. After that, MCF7 were treated with **2** 1.5 µM, **41** 5 µM, and K858 10 µM in a serum-free DMEM at cell density of 65,000/150 µL and then added to the upper chamber of insert. Medium with 10% of FBS was added to the lower chamber as a chemoattractant and the assay was performed after 48 h of treatment.

For cell migration assay, cells were treated and seeded directly in the upper chamber of insert, as previously described for invasion assay, and the medium with 10% of FBS was added to the lower chamber as a chemoattractant allowing cells to migrate; the assay was performed after 24 h of treatment.

At the established experimental times, remaining cells on the membrane top side were removed using a cotton swab, whereas invading or migrating cells were fixed with 5% of glutaraldehyde for 15 min and stained with crystal violet for 10 min at room temperature. Images were acquired with an inverted light microscope Leica Dmi1 (Leica Cambridge Ltd., Cambridge, UK Leica) provided with a camera Leica MC120 HD (Leica Cambridge Ltd., Cambridge, UK) for the acquisition of digital images. Images were analyzed by Leica Application Suite-X (LAS-X) image analysis software. The invasion and migration levels percentages were established by measuring the surface covered by crystal violet-stained cells.

### 2.6. Wound Healing

When MCF7 cells, seeded in a 6-well tissue culture plate, reached 70% confluence, cell monolayer was scraped with a p200 pipet tip and treated with **2** 1.5 µM, **41** 5 µM, and K858 10 µM. After 0, 24 and 48 h of treatment the images were taken with an inverted light microscope Leica Dmi1 (Leica Cambridge Ltd., Cambridge, UK Leica) provided with a camera Leica MC120 HD (Leica Cambridge Ltd., Cambridge, UK). The cut width was quantified by LAS-X image software (Leica Cambridge Ltd., Cambridge, UK) and expressed as Pixel.

### 2.7. Protein Extraction and Western Blot analysis

MCF7 cells were seeded in a 6-well plate at 250,000 cells/well density and treated with **2**, **41**, and K858, as mentioned before. After 24 and 48 h of treatment, cells were trypsinized, centrifuged, and harvested in cold PBS. Pellets were lysed in RIPA buffer containing inhibitors (Phenylmethylsulfonyl fluoride (PMSF) 100 µg/mL, Aprotinin 10 µg/mL, Leupeptin 50 µg/mL and Sodium Orthovanadate 1 mM (all purchased from Merck Life Science, Milan, Italy). A bicinchoninic acid assay (QuantiPro™ BCA Assay kit, Merck Life Science, Milan, Italy) was used to determine protein concentration following the manufacturer’s indications.

An amount of 20 µg of lysate were run on 8% and 12% (SDS)-polyacrylamide gel by electrophoresis and transferred to a nitrocellulose membrane, for each sample The membrane was blocked in 5% non-fat milk or 5% BSA, 10 mmol/L Tris-HCl pH 7.50, 100 mmol/L NaCl, 0.1% (*v*/*v*) Tween-20 and probed with mouse monoclonal anti-tubulin (dilution 1:5000), anti-Bcl-2 (dilution 1:100), anti-Bax (dilution 1:100), anti-HIF-1α (dilution 1:100), anti-MMP-9 (dilution 1:200), anti-PARP-1 (dilution 1:100), rabbit polyclonal anti-VEGF (dilution 1:200) antibody (Santa Cruz Biotechnology, Santa Cruz, CA, USA), rabbit monoclonal anti-caspase-3 (dilution 1:1000) antibody (Cell Signaling Technology, Danvers, MA, USA) and rabbit polyclonal anti-NF-kB p65 (dilution 1:500) antibody (Abcam, Cambridge, UK). After overnight incubation at 4 °C under gentle shaking, the membrane was probed with specific IgG horseradish peroxidase (HRP)-conjugated secondary antibodies (Calbiochem, Darmstadt, Germany). Immunoreactive bands were revealed by means of the ECL detection system (LiteAblot Extend Chemiluminescent Substrate, EuroClone S.p.A., Milan, Italy), then underwent a densitometric analysis. Densitometric values, expressed as Integrated Optical Intensity (IOI), were evaluated by a ChemiDoc™ XRS system and the QuantiOne 1-D analysis software (BIORAD, Richmond, CA, USA). Values were normalized on internal Tubulin values.

### 2.8. Detection of Apoptosis and Necrosis by Flow Cytometry

MCF7 cells were cultured in 6-well plates and underwent treatments as previously described, for 48 h. After having harvested supernatants, cells were washed once with PBS at room temperature. Then, cells were detached by trypsin, and supernatants were collected after centrifugation. Staining with annexin V and propidium iodide (PI) (eBioscience, Thermo Fisher Scientific, Waltham, MA, USA) was carried out in order to discriminate apoptotic and necrotic cells, according to the manufacturer’s instructions. Samples were probed in binding buffer and of annexin V FITC (197 μL +3 μL, respectively), in the dark, for 15 min at room temperature. After that, the volumes were doubled, and the cells were centrifuged and resuspended in binding buffer with PI. A CytoFlex flow cytometer (Beckman Coulter, Brea, CA, USA) was used to determine the fluorescence. A 530/30 bandpass filter was used to analyze FITC fluorescence (FL-1), and a 650 nm long pass filter for PI fluorescence (FL-3). Data were acquired (2 × 10^4^ events/sample) and analyzed by means of the CytExpert Software (Beckman Coulter, Brea, CA, USA). Viable cells percentage (annexin V^−^; PI^−^) were revealed in the lower left quadrant (unstained) of density plots, cells in apoptosis in lower right quadrant (annexin V^+^/PI^−^), cells in late apoptosis in upper right quadrant (annexin V^+^/PI^+^) and cells in necrosis in upper left quadrant (annexin V^−^/PI^+^).

### 2.9. Statistical Analysis

Data were statistically analyzed by GraphPad 7 software (San Diego, CA, USA) using ordinary one−way ANOVA followed by post hoc Tukey’s multiple comparison test.

## 3. Results

### 3.1. Effect of K858 and Its Analogues on MCF7 Cell Viability

**Figure 1:**
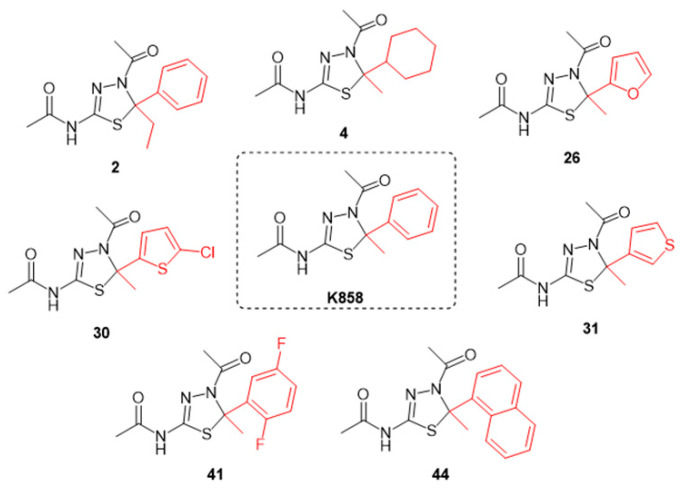
Molecular structures of seven thiadiazoline Eg5 inhibitors and their parent compound K858. In red, different substitutions on the C5.

With the aim of evaluating the capability of newly synthetized Eg5 inhibitors, namely **2**, **4**, **26**, **30**, **31**, **41** and **44**, to affect MCF7 viability, an initial screening, by means of MTT assay, was performed. These compounds are analogues of K858 presenting the same thiadiazoline core nucleus of K858 with different substituents at C5 (Figure 1).

**Figure 2:**
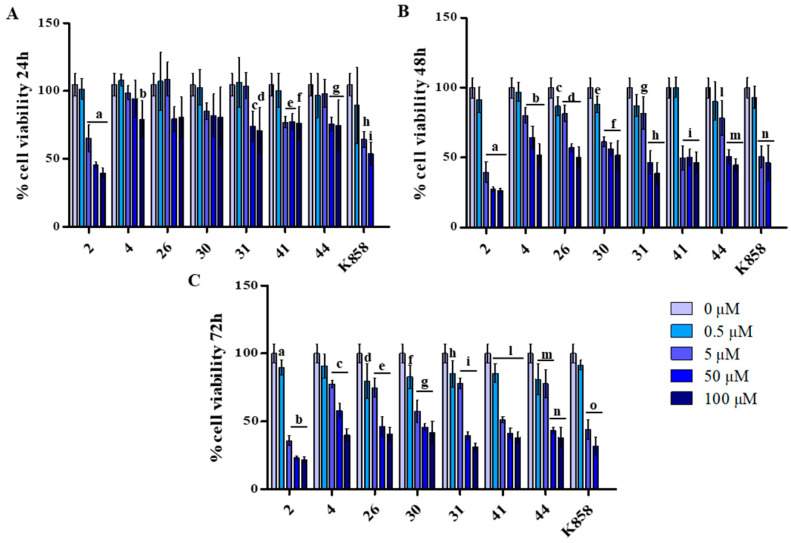
MTT assay on MCF7 cells exposed to compounds **2**, **4**, **26**, **30**, **31**, **41**, **44**, and K858 (from 0.5 to 100 μM) for 24, 48, and 72 h (A, B, and C, respectively). Metabolic activity was normalized with control cells treated with DMSO (0.2% as final concentration). DMSO: control vehicle. Data shown represent the mean ± SD of three independent experiments. (**A**) 2: a = *p* < 0.0001 0 µM vs. 5–50–100 µM; 4: b = *p* < 0.0017 0 µM vs. 100 µM; 31: c = *p* < 0.003 0 µM vs. 50 µM—d = *p* < 0.0003 0 µM vs. 100 µM; 41: e = *p* < 0.001 0 µM vs. 5–50 µM—f = *p* < 0.0001 0 µM vs. 100 µM; 44: g = *p* < 0.0028 0 µM vs. 50–100 µM; K858: h = *p* < 0.004 0 µM vs. 5 µM—i = *p* < 0.0004 0 µM vs. 50 µM. (**B**) 2: a = *p* < 0.0001 0 µM vs. 5–50–100 µM; 4: b = *p* < 0.0001 0 µM vs. 5–50–100 µM; 26: c = *p* < 0.01 0 µM vs. 0.5 µM—d = *p* < 0.0001 0 µM vs. 5–50–100 µM; 30: e = *p* < 0.01 0 µM vs. 0.5 µM—f = *p* < 0.0001 0 µM vs. 5–50–100 µM; 31: g = *p* < 0.01 0 µM vs. 5 µM—h = *p* < 0.0001 0 µM vs. 50–100 µM; 41: i = *p* < 0.0001 0 µM vs. 5–50–100 µM; 44: l = *p* < 0.001 0 µM vs. 5 µM—m = *p* < 0.0001 0 µM vs. 50–100 µM; K858: n = *p* < 0.0001 0 µM vs. 5–50 µM. (**C**) 2: a = *p* < 0.001 0 µM vs. 0.5 µM—b = *p* < 0.0001 0 µM vs. 5–50–100 µM; 4: c = *p* < 0.0001 0 µM vs. 5–50–100 µM; 26: d = *p* < 0.001 0 µM vs. 0.5 µM—e = *p* < 0.0001 0 µM vs. 5–50–100 µM; 30: f = *p* < 0.001 0 µM vs. 0.5 µM—g = *p* < 0.0001 0 µM vs. 5–50–100 µM; 31: h = *p* < 0.001 0 µM vs. 0.5 µM—i = *p* < 0.0001 0 µM vs. 5–50–100 µM; 41: l = *p* < 0.0001 0 µM vs. 0.5–5–50–100 µM; 44: m = *p* < 0.001 0 µM vs. 0.5–5 µM—n = *p* < 0.0001 0 µM vs. 50–100 µM; K858: o = *p* < 0.0001 0 µM vs. 5–50 µM.

The compounds were tested on MCF7 at 0.5, 5, 50, and 100 µM, while K858 was tested at 0.5, 5, and 50 µM, all for 24 h (Figure 2A), 48 h (Figure 2B), and 72 h (Figure 2C).

After 24 h of treatment at the highest dose administered (100 µM) for compounds **4** and **26**, the recorded cell viability percentage is of 80%, approximately. After 48 h of treatment, the viability rate recorded at 50 µM still is higher with respect to that recorded with other compounds and the cell viability percentage reaches 50% only after 72 h of treatment. Compounds **31** and **44** lead to record viability levels higher than 50% after 24 h of treatment (approximately 77% at 50 µM), while after 48 and 72 h they significantly affect cell viability leading to measure percentages lower than 50%. Compound **2** shows a different profile as, after 24 h of treatment and at the lower dose (5 µM) administered it determines a reduction of cell viability at approximately 65%, reaching 39% and 35% after 48 h and 72 h of treatment, respectively. Compounds **30** and **41** exhibit a similar trend: after 24 h of treatment at the lower dose administered (5 µM) the recorded cell viability level is about 80%, after 48 and 72 h it reaches 50%, with a major extent for **41**.

**Figure 3:**
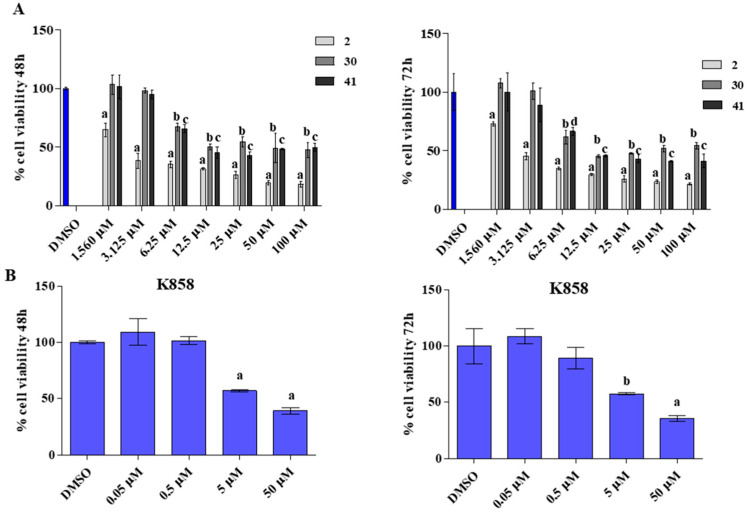
MTT test on MCF7 cells exposed to different concentrations of **2**, **41**, **30** (**A**) and to different concentrations of K858 (**B**) for 48 and 72 h. Metabolic activity was normalized with DMSO-treated cells (0.2% as final concentration). DMSO: control vehicle. Data shown represent the mean ± SD of three independent experiments. (**A**) a = *p* < 0.0001 DMSO vs. and all tested concentration of 2 at both time points; b = *p* < 0.0001 DMSO vs. doses from 6.25 µM to 100 µM of 30 at both time points; c = *p* < 0.0001 DMSO vs. doses from 6.25 µM to 100 µM of 41 at 48 h; c = *p* < 0.0001 DMSO vs. doses from 12.5 µM to 100 µM of 41 at 72 h; d = *p* < 0.01 DMSO vs. 6.25 µM of 41 at 48 h. (**B**) a = *p* < 0.0001 DMSO vs. 5 µM–50 µM of K858 at 48 h; a = *p* < 0.0001 DMSO vs. 5 µM of K858 at 72 h; b = *p* < 0.001 DMSO vs. 5 µM of K858 at 72 h.

Based on these preliminary screening results, **2**, **30,** and **41** were selected for further analyses on MCF7. Firstly, a new viability test was performed in order to establish a subtoxic concentration to be applied in future experiments. MCF7 were treated with **2**, **30,** and **41** at doses ranging from 1.560 µM to 100 µM, whereas K858 was administered at 0.05, 0.5, 5, and 50 µM, all for 48 and 72 h (Figure 3). These three compounds were also characterized by good gastrointestinal absorption, discrete aqueous solubility, and no attitude to act as PAINS (Pan-Assay INterfering assayS) according to in silico evaluation using commercially available web tools.

After 48 and 72 h of treatment with **2** at 1.560 µM MCF7 cell viability is of 60% approximately, while after 48 and 72 h at 3.125 µM cell viability percentage reaches 45% (Figure 3A). Compounds **30** and **41** lead to record a cell viability level of about 60 when administered at 6.25 µM after both time points (Figure 3A). These results are compared with the results of the parent compound K858 which determines a cell viability rate of 50% at 5 µM after 48 and 72 h of treatment (Figure 3B). Since **2** and **41** were already tested in our previous work [24,25] on AGS gastric adenocarcinoma cell line evidencing their capability to counteract cells proliferation and invasiveness, the same molecules are kept for further investigations, excluding compound **30**, in order to explore similarities or differences in the effect exerted on MCF7 cell line. Thus, based on the results obtained from the MTT test, 1.5 µM for **2** and 5 µM for **41** are chosen as subtoxic concentrations for future experiments, whereas 10 µM as subtoxic concentration is chosen for K858, as reported by De Iuliis and co-workers [20] and confirmed by our cell viability results.

### 3.2. Effect of 2, 41, and K858 on non-Tumoral Cells Viability

**Figure 4:**
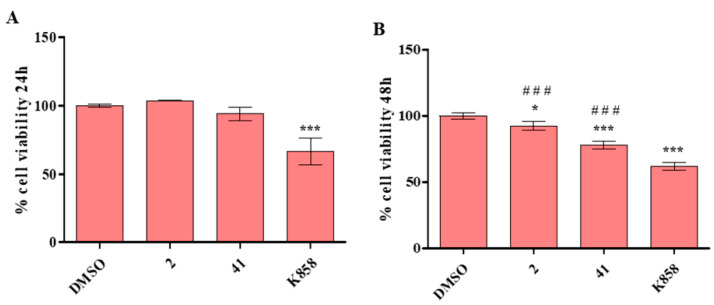
MTT test on HGF cells exposed to 2 (1.5 μM), 41 (5 μM), and K858 (10 μM) for 24 (**A**) and 48 h (**B**). Metabolic activity was normalized to DMSO-treated cells (0.2% as final concentration). DMSO: control vehicle. Data shown represent the mean ± SD of three independent experiments. (**A**): *** *p* < 0.0001 DMSO vs. K858. (**B**): *** *p* < 0.0001 DMSO vs. Eg5 inhibitors; * *p* < 0.01 DMSO vs. Eg5 inhibitors; ^###^ *p* < 0.0001 K858 vs. **2** and **41**.

With the aim of evaluating the effect of Eg5 inhibitors at the selected concentrations on non-tumoral cells, **2**, **41,** and K858 were administered to HGFs and cell viability percentages, obtained from the MTT test, were read. After 24 h of treatment, K858 at 10 µM affects HGF viability in a significant manner: 60% approximately compared to untreated cells (100%), to **2** at 1.5 µM (100%), and to **41** at 5 µM (94%) (Figure 4A).

After 48 h of treatment, a similar trend is evidenced: K858 decreases cell viability in a statistically significant manner (61%) compared to DMSO (100%), to **2** (92%), and to **41** (77%). Compounds **2** and **41** induce a significant cell viability reduction compared to DMSO (Figure 4B).

### 3.3. Effect of 2, 41, and K858 on MCF7 Cytotoxicity

**Figure 5:**
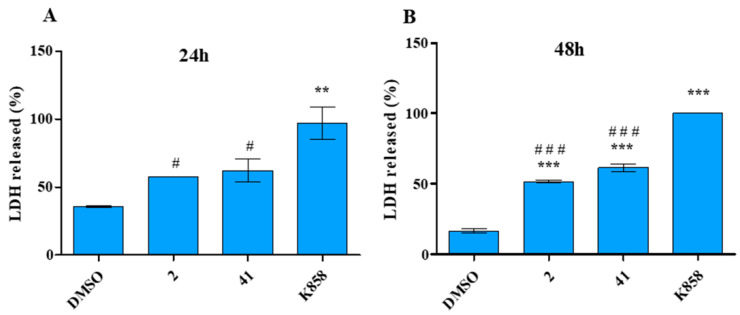
Cytotoxicity assay (LDH assay) of MCF7 cells treated with vehicle (DMSO), 2 (1.5 μM), 41 (5 μM), and K858 (10 μM) for 24 (**A**) and 48 h (**B**). (**A**): ** *p* < 0.0051 DMSO vs. K858; ^#^ *p* < 0.05 2 and 41 vs. K858. (**B**): *** *p* < 0.0001 DMSO vs. Eg5 inhibitors; ^###^ *p* < 0.001 K858 vs. 2 and 41. LDH leakage is reported as percentage. Data are presented as the mean ± SD of three separate experiments.

The MCF7 cytotoxicity was then evaluated by measuring the released LDH level within the culture medium, after 24 and 48 h of treatment with **2**, **41,** and K858 at the established concentrations. After 24 h, K858 determines a statistically significant increase in LDH release with respect to **2**, **41** and to untreated cells (LDH released: DMSO = 35.78%, **2** = 57.60%, **41** = 62.14%, K858 = 97.13%) (Figure 5A).

After 48 h of treatment, the trend is maintained as K858 induces a higher significant LDH release (100%) with respect to untreated cells (16.6%), **2**-treated (51.6%), and **41**-treated (61.5%) cells; moreover, after 48 h cells exposed to **2** and to **41** disclose a significant increase in LDH release with respect to untreated cells (Figure 5B).

### 3.4. Effect of Eg5 Inhibitors on MCF7 Cell Invasion, Migration and on NF-kB, MMP-9, HIF-1α, and VEGF Proteins Expression

**Figure 6:**
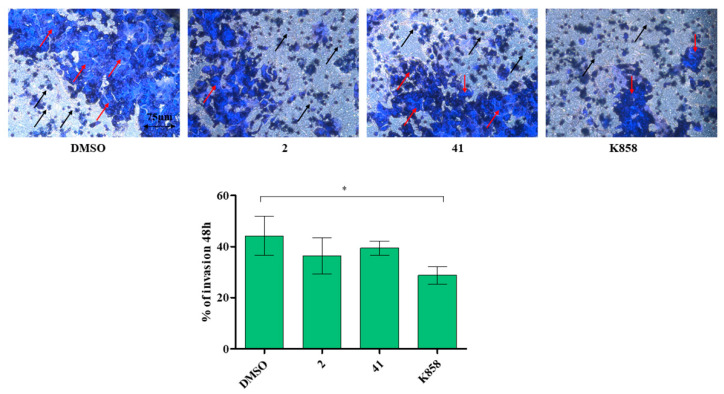
Transwell invasion assay of MCF7 in the presence of compounds **2** (1.5 μM), **41** (5 μM), and K858 (10 μM). Images represent invading cells after crystal violet staining. Histogram represents densitometric analysis obtained by quantifying thresholded area for violet color in 10 fields for each of three slides per sample. Magnification 20×. Dark blue represents stained invasive cells, (red arrows), light blue surface represents the insert with pores (black arrows). * *p* < 0.01. DMSO: control vehicle. Data are presented as mean ± SD.

A specific assay was carried out to investigate MCF7 invasion capability after 48 h of treatment with **2**, **41,** and K858. K858 discloses a statistically significant reduction in the cell invasion percentage (28.7%) compared to untreated cells (44%), while **2** and **41** do not lead to significant cell invasion % reduction with respect to DMSO (36% and 39%, respectively) (Figure 6).

**Figure 7:**
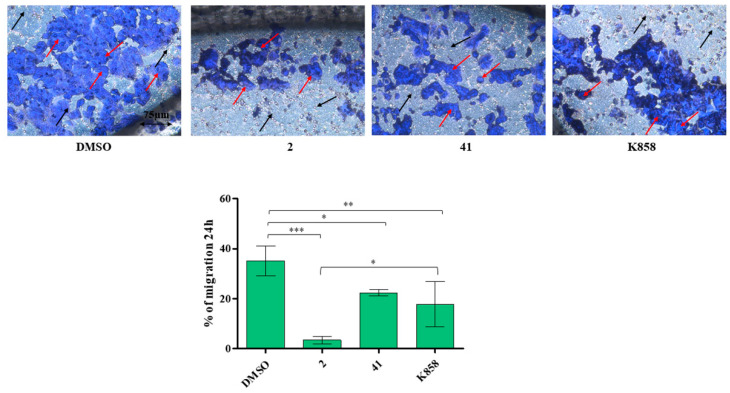
Transwell migration assay of MCF7 treated with compounds **2** (1.5 μM), **41** (5 μM), and K858 (10 μM). Images represent migrated cells after crystal violet staining. Histogram represents densitometric analysis obtained by quantifying thresholded area for violet color in 10 fields for each of three slides per sample. Magnification 20×. Dark blue represents stained invasive cells (red arrows), light blue surface represents the insert with pores (black arrows). *** *p* < 0.0001; ** *p* < 0.001; * *p* < 0.01. DMSO: control vehicle. Data are presented as mean ± SD.

A migration and a wound healing assay, after 24 h and after 24 h and 48 h, respectively, were performed on MCF7 to evaluate **2**, **41,** and K858 capability in controlling cell migration. After administration of **2**, **41,** and K858 at the established concentrations for 24 h, untreated MCF7 migration is of 35% approximately, while after **41** and K858 treatment the migration is significantly reduced with respect to DMSO (22% and 17%, respectively). Further, **2** shows a strong significant capability to suppress cell migration with respect to untreated cells (3%); both new Eg5 inhibitors **2** and **41** are able to reduce MCF7 cell migration in a statistically significant manner compared to the parent compound K858 (Figure 7).

**Figure 8:**
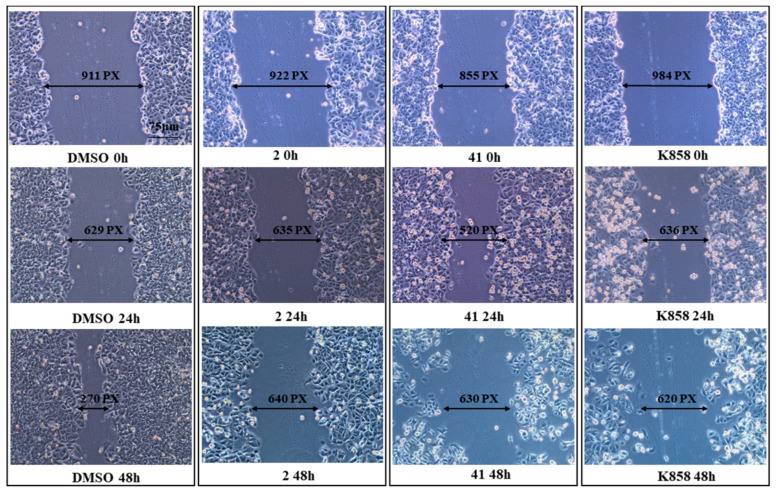
Scratch wound healing assay performed in MCF7 cells treated with compounds **2** (1.5 μM), **41** (5 μM) and K858 (10 μM). Images were taken at 0, 24, and 48 h after the confluent monolayer of cells was wounded. DMSO: control vehicle. The most representative of three separate experiments is shown. Magnification 20×.

These results are confirmed by wound healing assay: after 24 h, untreated cells show a reduction of cut width, with respect to 0 h, and after 48 h this cut is almost completely closed. A similar reduction of cut width is recorded in MCF7 treated with **2**, **41,** and K858 after 24 h of treatment. Conversely, after 48 h the cut width of MCF7 exposed to all Eg5 inhibitors does not show any narrowing compared to the cut width of cells exposed to DMSO (Figure 8).

**Figure 9:**
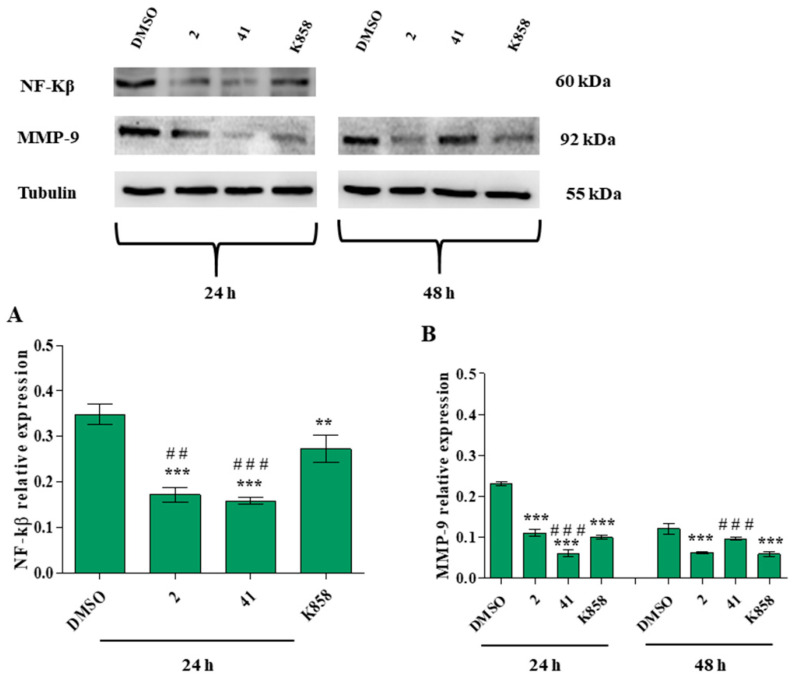
Nuclear factor kappa-B (NF-kB) (**A**) and matrix metalloproteinase-9 (MMP-9) (**B**) protein expression levels in MCF7 cell line treated with 2 (1.5 μM), 41 (5 μM) and K858 (10 μM) for 24 h (for NF-kB) and for 24, 48 h (for MMP-9). DMSO: control vehicle. Data are reported as means ± SD of three independent experiments. Tubulin is used as a loading control. The bar graph displays densitometric values normalized on loading control. *** *p* < 0.0001 DMSO vs. Eg5 inhibitors; ** *p* < 0.001 DMSO vs. Eg5 inhibitors; ^###^ *p* < 0.0001 K858 vs. 2 and 41; ^##^ *p* < 0.001 K858 vs. 2 and 41.

To better clarify the signaling cascades recruited by **2**, **41,** and K858 in cell invasion and migration processes, nuclear factor kappa-B (NF-kB) and matrix metalloproteinase-9 (MMP-9) expression were evaluated by means of western blot analysis. As NF-kB is a nuclear transcription factor, its expression was measured earlier (24 h) than the downstream molecule. After 24 h of treatment, all tested Eg5 inhibitors reduce NF-kB expression with respect to untreated cells; furthermore, **2** and **41** determine a statistically significant protein expression decrease compared to the parent compound K858 (Figure 9A).

A similar trend is recorded for MMP-9: after 24 h of treatment **2**, **41** and K858 significantly reduce the MMP-9 expression compared to DMSO; conversely, **41** decreases MMP-9 expression levels compared to K858. After 48 h of treatment, **2** and K858 decrease MMP-9 expression in a statistically significant manner compared to untreated cells and, in addition, K858 induces a significant reduction in protein expression compared to **41** (Figure 9B).

**Figure 10:**
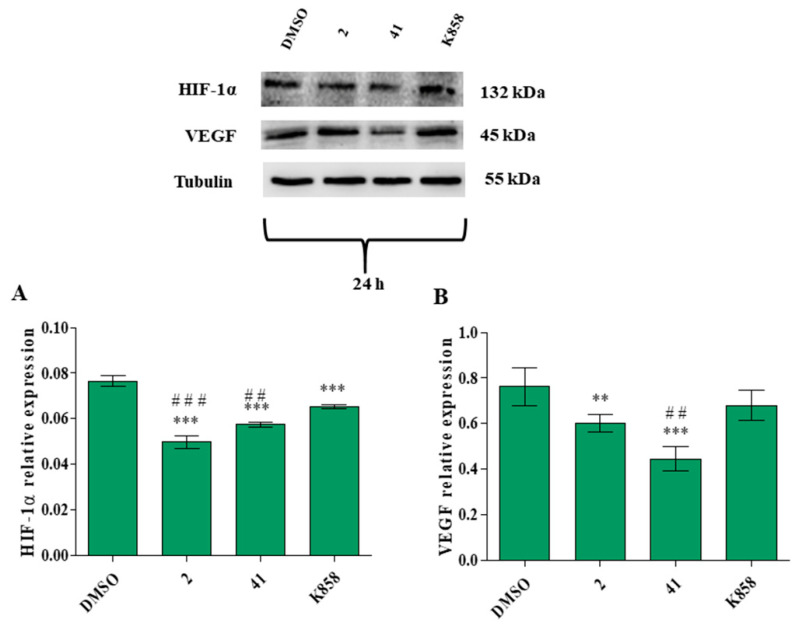
Hypoxia inducible factor 1α (HIF-1α) (**A**) and vascular endothelial growth factor (VEGF) (**B**) protein expression levels in MCF7 cell line treated with 2 (1.5 μM), 41 (5 μM) and K858 (10 μM) for 24 h. DMSO: control vehicle. Data are reported as means ± SD of three independent experiments. Tubulin is used as a loading control. The bar graph displays densitometric values normalized on loading control. (**A**): *** *p* < 0.0001 DMSO vs. Eg5 inhibitors; ^###^ *p* < 0.0001 K858 vs. 2 and 41; ^##^ *p* < 0.001 K858 vs. 2 and 41. (**B**): *** *p* < 0.0002 DMSO vs. Eg5 inhibitors; ** *p* < 0.002 DMSO vs. Eg5 inhibitors; ^##^ *p* < 0.002 K858 vs. 2 and 41.

Considering that the invasion and migration capabilities of cancer cells are closely related to the angiogenic process, hypoxia-inducible factor 1α (HIF-1α) and vascular endothelial growth factor (VEGF) proteins expression was also estimated after 24 h of treatment with Eg5 inhibitors by means of western blot analysis. All compounds provoke a strong and significant reduction of HIF-1α expression with respect to control; moreover, **2** and **41** significantly decrease HIF-1α expression with respect to K858 (Figure 10).

After 24 h of treatment VEGF expression levels appear significantly reduced in the presence of **2** and **41** with respect to control, while no significant modification is evidenced after exposure to K858; **41** significantly decreases VEGF expression with respect to K858 (Figure 10B).

### 3.5. Evaluation of MCF7 Apoptosis and Necrosis Induction by 2, 41, and K858 Treatment

**Figure 11:**
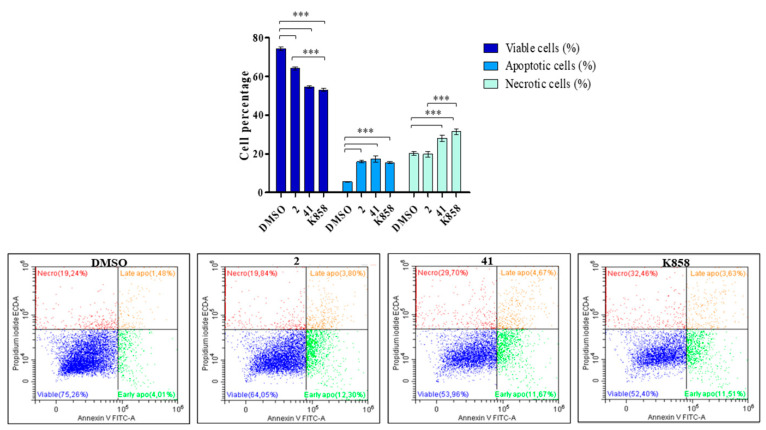
Analysis of apoptosis and necrosis occurrence in MCF7 cells treated with 2 (1.5 μM), 41 (5 μM), and K858 (10 μM) for 48 h. Percentages of cells, analyzed by flow cytometry, after staining with annexin V-FITC and propidium iodide (PI), are shown. DMSO: control vehicle. Bars represent the percentage of viable cells (unstained), total apoptotic cells (annexin V-positive and annexin V+PI-positive), and necrotic cells (PI-positive) as medians ± SD calculated from three independent experiments. *** *p* < 0.0001. Representative dual-parameter fluorescence density dot plots at 48 h are displayed in the lower panel.

After exposure to **2**, **41,** and K858 for 48 h, apoptosis and necrosis occurrence were evaluated by flow cytometry. Untreated cells appear viable, being negative for both annexin V and PI, with about 75% of cell viability. Conversely, all compounds induce a statistically significant reduction in the percentage of viable cells with respect to DMSO, with a major extent for MCF7 treated with **41** (54%) and K858 (52%). The percentage of apoptotic cells is significantly augmented when MCF7 are exposed to **2** (16%), **41** (17%), and K858 (15%) with respect to untreated cells (6%); **41** and K858 also induce an increase in necrotic cells (28% and 31%, respectively), in a statistically significant manner with respect to control (Figure 11).

**Figure 12:**
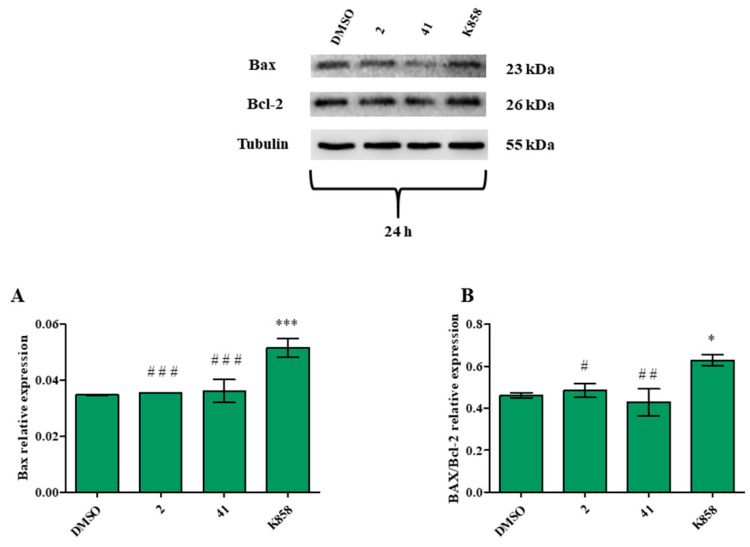
Bax and Bcl-2 protein expression levels in MCF7 cell line treated with 2 (1.5 μM), 41 (5 μM), and K858 (10 μM) for 24 h. DMSO: control vehicle. Data are reported as means ± SD of three independent experiments. Tubulin is used as a loading control. (**A**): The bar graph displays Bax densitometric values normalized on loading control. *** *p* < 0.0001 DMSO vs. K858; ^###^ *p* < 0.0001 K858 vs. 2 and 41. (**B**): The bar graph displays densitometric values expressed as mean ± SD of Bax/Bcl-2 ratio normalized on loading control. * *p* < 0.05 DMSO vs. K858; ^##^ *p* < 0.0074 K858 vs. 41; ^#^ *p* < 0.05 K858 vs. 2.

Therefore, the expression of a pro-apoptotic and an anti-apoptotic protein, represented by Bax and Bcl-2, respectively, was measured through a western blot after 24 h of treatment. Bax expression is statistically increased after treatment with K858 compared to DMSO and to **2** and **41** (Figure 12A); the same trend is recorded for Bax/Bcl-2 ratio (Figure 12B).

**Figure 13:**
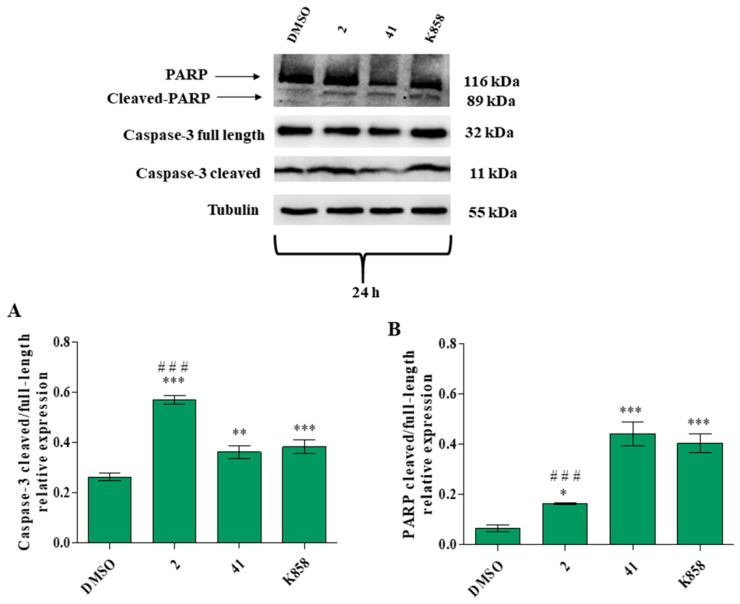
Caspase-3 and poly(ADP-ribose) polymerase (PARP) protein expression levels in MCF7 cell line treated with 2 (1.5 μM), 41 (5 μM) and K858 (10 μM) for 24 h. DMSO: control vehicle. Data are reported as means ± SD of three independent experiments. Tubulin is used as a loading control. (**A**): The bar graph displays caspase-3 cleaved/full-length ratio densitometric values normalized on loading control. *** *p* < 0.0001; ** *p* < 0.001. (**B**): The bar graph displays PARP cleaved/full-length ratio densitometric values normalized on loading control. *** *p* < 0.0001 DMSO vs. Eg5 inhibitors; ** *p* < 0.001 DMSO vs. Eg5 inhibitors; * *p* < 0.01 DMSO vs. Eg5 inhibitors; ^###^ *p* < 0.0001 K858 vs. 2 and 41.

Lastly, in order to shed light and better clarify the apoptosis and necrosis occurrence during treatment with novel Eg5 inhibitors, caspase-3, and poly(ADP-ribose) polymerase (PARP) expression levels were also quantified. Compound **2** statistically increases the caspase-3 cleaved/full-length ratio with respect to untreated cells and to K858. Compounds **41** and K858 provoke an augmentation of caspase-3 cleaved/full-length ratio compared to control, while there is not a statistically significant difference between **41** and K858 (Figure 13A).

In contrast, compound **2** induces a statistically significant increase in PARP cleaved/full-length ratio in comparison to untreated cells, whereas **41** and K858 provoke a significant increase in PARP cleaved/full-length ratio compared to DMSO. In addition, the parent compound K858 increases the expression of PARP in a statistically significant manner with respect to **2** (Figure 13B). All original images of western blot are available in the Supplementary material (Figures S1–S11)

## 4. Discussion

Considering the necessity of discovering new molecules to overcome side effects and tumor resistance to common anticancer drugs and based on the possibility to find new molecular targets to treat patients, researchers spent efforts on the purpose of finding new therapies including innovative compounds. With this aim, new targets have been explored, among them, kinesin Eg5, which has been demonstrated to be involved in several widely diffused cancers [11,12,13,14,15].

Seven newly synthetized Eg5 inhibitors were already tested on gastric adenocarcinoma cell line and two of them, **2** and **41**, were chosen for their promising in vitro effects in counteracting tumor cell viability and in negatively modulating angiogenic cascade [24,25]. Based on these results on gastric adenocarcinoma and since Eg5 has been reported to be overexpressed also in BC [17], kinesin Eg5 inhibitors, along with the parent compound K858, were tested on MCF7 breast adenocarcinoma cell line. As already found in our previous work [24], compounds **2** and **41** were selected from a wider panel for their capability to counteract MCF7 viability at lower concentrations, thus, 1.5 µM and 5 µM, for **2** and **41**, respectively, were established as subtoxic concentrations. Conversely, based on both what was reported in the scientific literature [20] and on our results, the parent compound K858 required a higher concentration, 10 µM, to obtain the same effect in terms of MCF7 viability reduction. This concentration is probably also responsible for the higher MCF7 cytotoxicity and percentage of dead healthy cells registered for K858 and not for **2** and **41**. These preliminary findings underline the possibility to administer **2** and **41** at lower doses to preserve healthy cell viability.

The main cause of BC mortality is metastasis formation: in this process cancer cells spread outside the primary tumor mass to form secondary tumoral sites, far from the origin point [28]. During this process cell invasion represents the first step, in which tumor cells penetrate the basement barrier and enter nearby tissues. After the invasion, cancer cells’ migration through the extracellular matrix (ECM) represents the second critical stage [29]. Previous works already reported that in different in vitro models, Eg5 inhibition could represent a valid strategy to counteract cancer cell invasion and migration [30]. Our experimental model results disclose the K858 capability to inhibit MCF7 cell invasion, thus validating the same feature previously demonstrated against head and neck squamous cancer cells [31] highlighting an appreciable K858 ability to reduce cell migration. Compounds **2** and **41** have already evidenced a strong effect in counteracting gastric adenocarcinoma cell migration [24], especially when **2** was combined with the hesperidin polyphenol [25]. This aspect was corroborated in MCF7 in which, in addition, the capability of **2** to control cell migration markedly exceeds K858 confirming the active role of Eg5 inhibition in preventing the key passages of metastasis formation.

The activity of matrix metalloproteinases (MMPs) is known to be crucial for cancer cell spread, in particular, MMP-9 triggers the degradation of ECM essential proteins, such as type IV collagen, allowing the spreading of cancer cells [32]. Previous reports have evidenced that K858 inhibits MMP-9 expression in human glioblastoma cells [33]. This finding is supported by our experimental model results, in which K858 is shown to reduce MMP-9 expression. Interestingly, the downregulation of MMP-9 expression is enhanced in presence of **2** and **41** with respect to K858. Several papers demonstrated that, in experimental tumor models among which breast cancer is included, a correlation between NF-kB and MMP-9 exists [34,35,36]. In detail, the transcriptional factor NF-kB can translocate into the nucleus activating MMP-9 gene transcription. Considering that MMP-9 expression markedly reflects NF-kB levels it is likely to suppose that novel Eg5 inhibitors can counteract MMP-9 expression through NF-kB inhibition even if further studies of upstream NF-kB silencing are needed to confirm this hypothesis

The degradation of ECM, led by MMP-9, speeds up the angiogenic process [37], essential for tumor mass growth, by promoting the release of downstream angiogenic factors in the tumor microenvironment, such as VEGF [38]. In fact, it is widely recognized that MMP-9 is synthesized by cancer cells regulating VEGF release promoting angiogenesis and spread of metastasis [39]. Conversely, MMP-9 inhibition leads to a reduction of endothelial cell invasion [40]. Furthermore, a correlation between VEGF and Eg5 has been already demonstrated: after human recombinant VEGF administration in endothelial cells, the gene encoding for Eg5 protein, namely KIF11, appeared upregulated [41]. Our results clearly confirm both these points. In fact, **2** and **41** negatively modulate VEGF expression, more than K858, as already demonstrated in the gastric adenocarcinoma model [25]. In parallel, the comparable levels of MMP-9 and VEGF lead to assume that a correlation between the two molecules could exist.

During the tumor mass growth, the oxygen supply is often poor, especially at the center of the mass, thus generating hypoxic regions. Cell survival in hypoxic conditions requires the activation of survival pathways recruiting the HIF-1α transcription factor [42] which, in turn, is involved in the angiogenetic process by transcriptionally activating several factors and their receptors, such as VEGF [43]. Our model demonstrated that kinesin inhibitors are able to downregulate HIF-1α expression. Taken together these results revealed that **2** and **41** kinesin inhibitors, better than their parent compound K858, negatively control VEGF activation by simultaneously acting both on MMP-9 and on HIF-1α, thus significantly reducing the possibility of VEGF recruitment. The results regarding the Eg5 inhibitors’ capability to counteract cell migration, invasion, and angiogenic pathways recruitment led us to focus our attention not only on the mitotic properties of Eg5 but also on the non-mitotic activities of this protein. In fact, among the non-mitotic functions, it has been proposed that Eg5 could also function as a movable molecular bridge between microtubules and ribosomes to improve the effectiveness of polypeptide synthesis [44] and also the transport of secretory proteins from the Golgi complex to the cell surface [45]. Thus, it could be argued that, through Eg5 inhibition, a block of the synthesis and of the transport of crucial proteins involved in cell migration and invasion occurs, as demonstrated also in our experimental model.

The ability of K858, **2,** and **41** to induce apoptosis, because of Eg5 inhibition and mitotic process failure, has been already reported in the scientific literature and by our group [20,24,31,33,46]. This ability to induce apoptosis is not an exclusive prerogative of K858 and its analogs: several compounds, belonging to different chemical classes of Eg5 inhibitors, such as monastrol, ispinesib, filasenib, etc., are able to trigger apoptotic cascade in many types of cancer models [47,48,49]. All these classes of Eg5 inhibitors are able to bind to an allosteric site located in the motor domain, formed by helix α2, loop L5, and helix α3. The present results are in accordance with previous findings: Eg5 inhibitors appeared to induce MCF7 apoptosis, as demonstrated both by the annexin PI results and by the increased caspase-3 and PARP levels. The high levels of Bax expression recorded after K858 treatment lead to suppose that the parent compound could activate the intrinsic apoptotic pathway, whereas **2** and **41** recruit alternative molecular signaling. In particular, it is likely that **2** triggers the extrinsic apoptotic pathway, as it provokes a caspase-3 activation without Bax recruitment; conversely, **41** seems to activate the necrotic pathway, as suggested by annexin PI results and by the higher level of PARP.

## 5. Conclusions

These findings shed light on two points. Firstly, they underline that kinesin Eg5 represents a valid and alternative target for advanced cancer therapies. Secondly, among thiadiazoline-based Eg5 inhibitors, **2** and **41**, simultaneously modulating several key processes of breast cancer spread and progression, such as cell proliferation, invasiveness, migration, ECM remodeling, and angiogenesis, along with apoptosis or necrosis occurrence, represent a new drug strategy to control breast cancer. As these are preliminary results derived from newly synthesized molecules, further detailed studies are needed to deepen the molecular mechanisms underlying Eg5 inhibitors administration and to aim at in vivo trials.

## Data Availability

The data that support the findings of this study are contained within the article and available from the corresponding author.

## Supplementary Materials

The following are available online at https://www.mdpi.com/article/10.3390/biology11101450/s1, Figure S1: western blot of HIF-1α at 24 h, Figure S2: western blot of VEGF at 24 h, Figure S3: western blot of BAX at 24 h, Figure S4: western blot of BCL-2 at 24 h, Figure S5: western blot of caspase-3 cleaved, Figure S6: western blot of caspase-3 full length, Figure S7: western blot of PARP at 24 h, Figure S8: western blot of MMP9 at 24 and 48 h, Figure S9: western blot of NF-kB at 24 h, Figure S10: western blot of tubulin at 24 h, Figure S11: western blot of tubulin at 48 h.
