# Supplementary figures and images for: Kinesin Eg5 Selective Inhibition by Newly Synthesized Molecules as an Alternative Approach to Counteract Breast Cancer Progression: An In Vitro Study
Source: Biology (Basel). 2022 Oct 2;11(10):1450. doi: 10.3390/biology11101450 (PMC9598199; doi:10.3390/biology11101450)

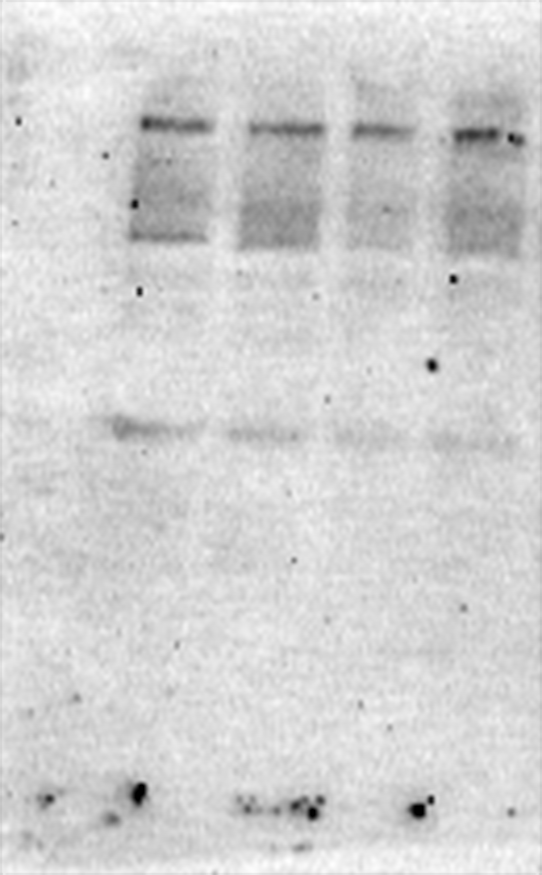

Supplement: Supplementary file 1 [file biology-11-01450-s001.zip › Figure S1.tif]

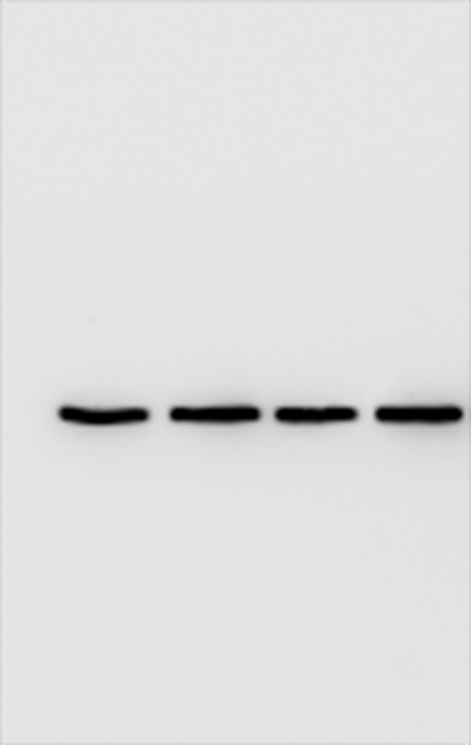

Supplement: Supplementary file 1 [file biology-11-01450-s001.zip › Figure S10.tif]

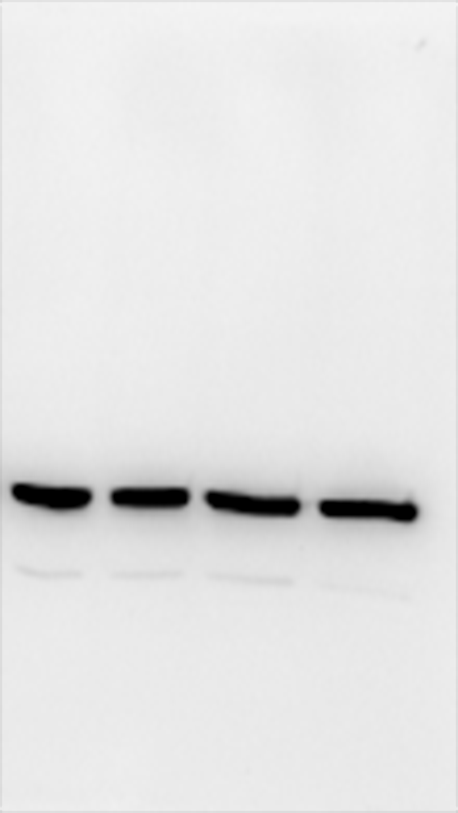

Supplement: Supplementary file 1 [file biology-11-01450-s001.zip › Figure S11.tif]

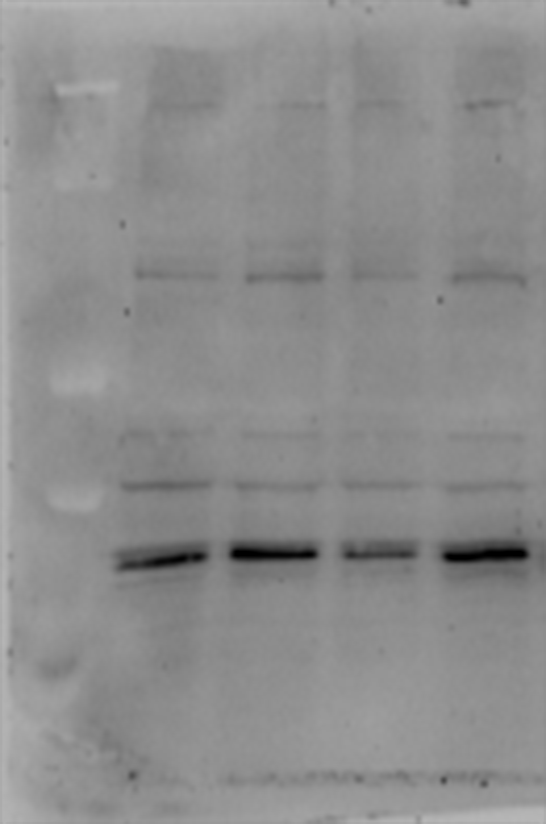

Supplement: Supplementary file 1 [file biology-11-01450-s001.zip › Figure S2.tif]

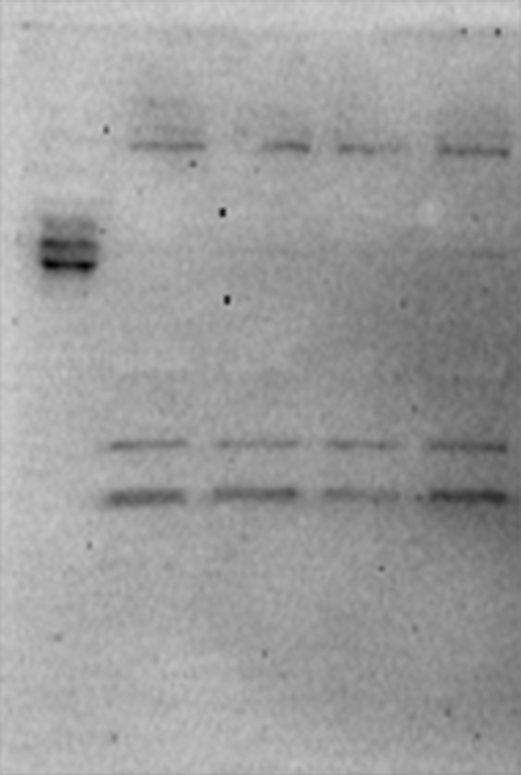

Supplement: Supplementary file 1 [file biology-11-01450-s001.zip › Figure S3.tif]

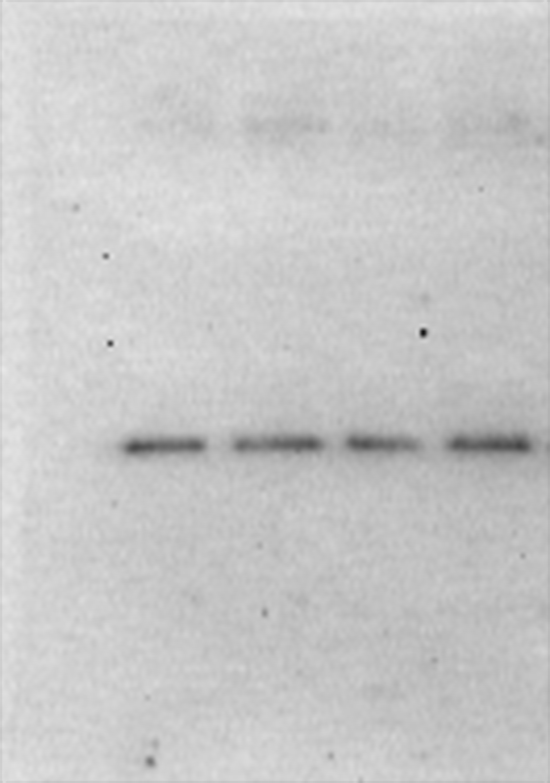

Supplement: Supplementary file 1 [file biology-11-01450-s001.zip › Figure S4.tif]

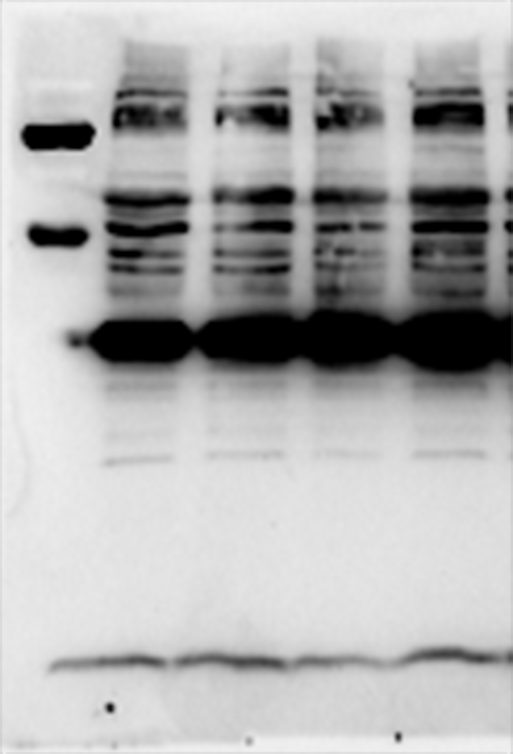

Supplement: Supplementary file 1 [file biology-11-01450-s001.zip › Figure S5.tif]

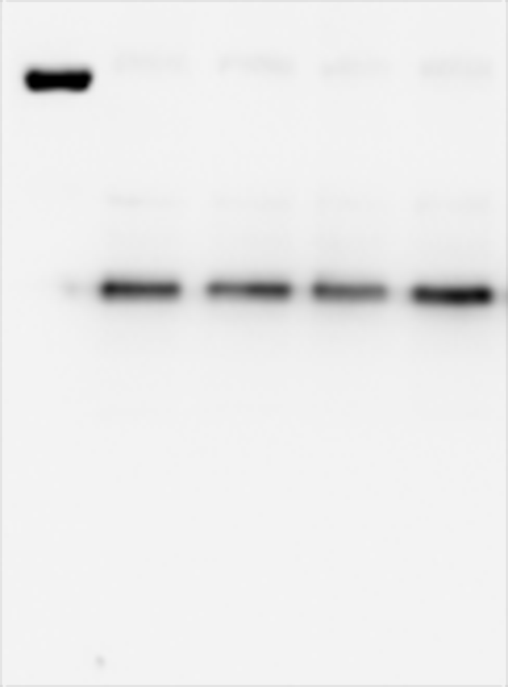

Supplement: Supplementary file 1 [file biology-11-01450-s001.zip › Figure S6.tif]

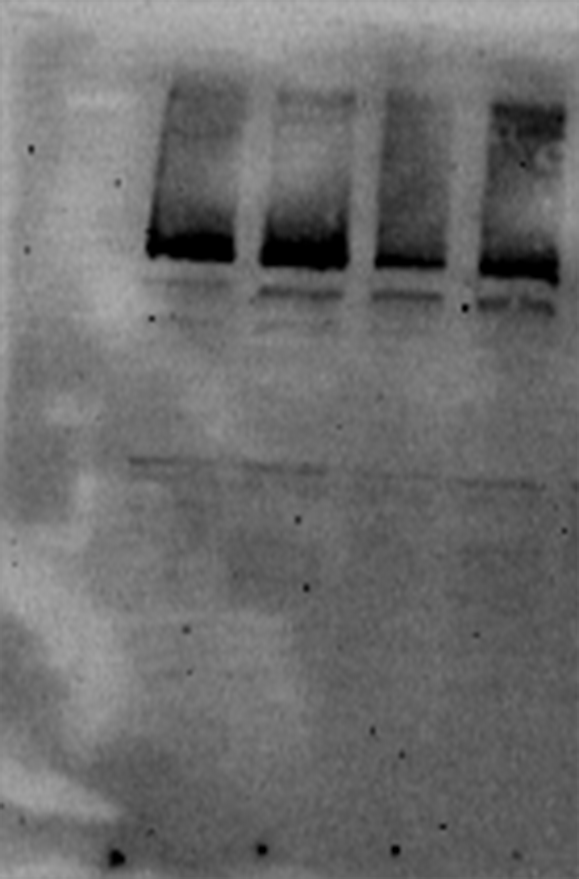

Supplement: Supplementary file 1 [file biology-11-01450-s001.zip › Figure S7.tif]

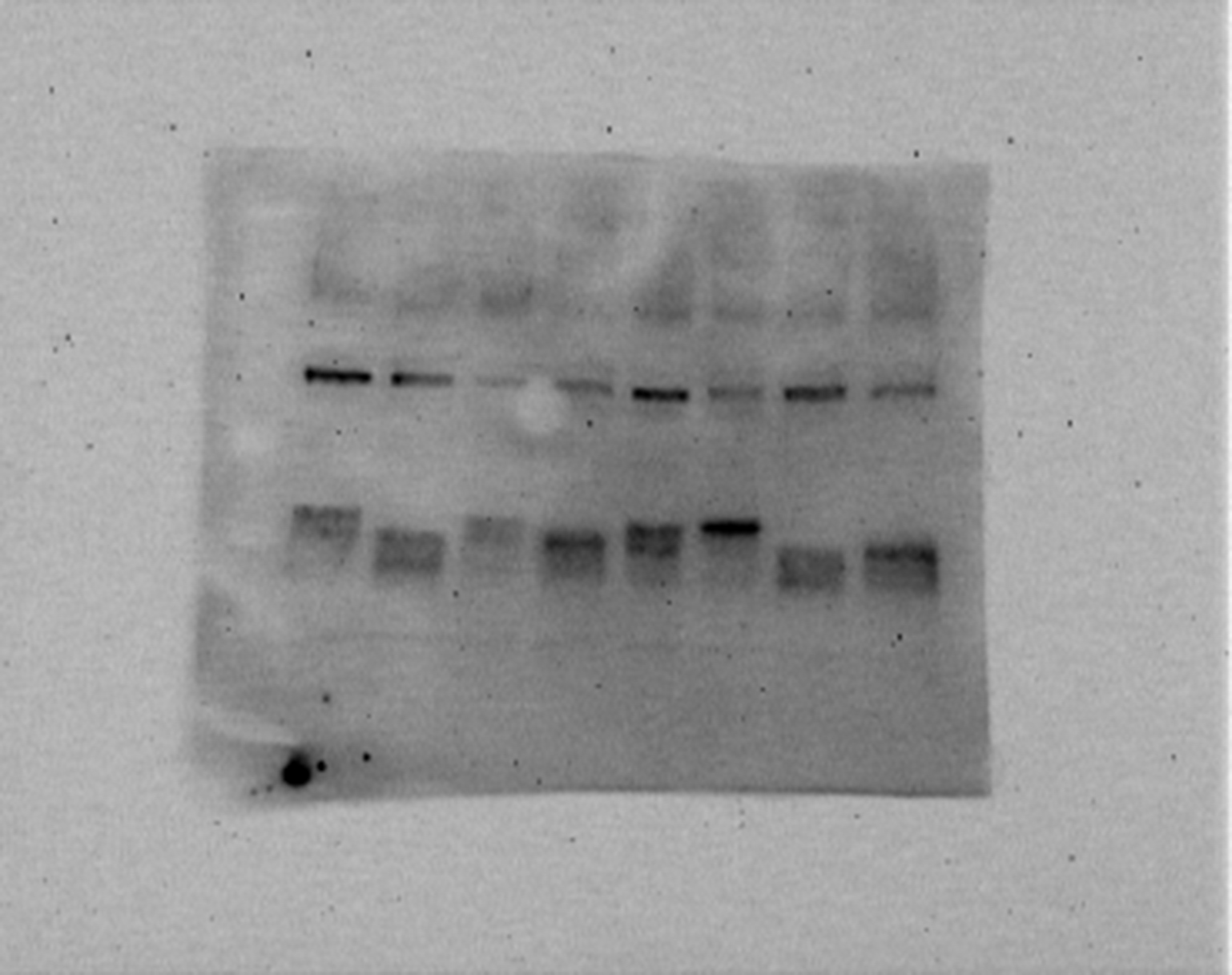

Supplement: Supplementary file 1 [file biology-11-01450-s001.zip › Figure S8.tif]

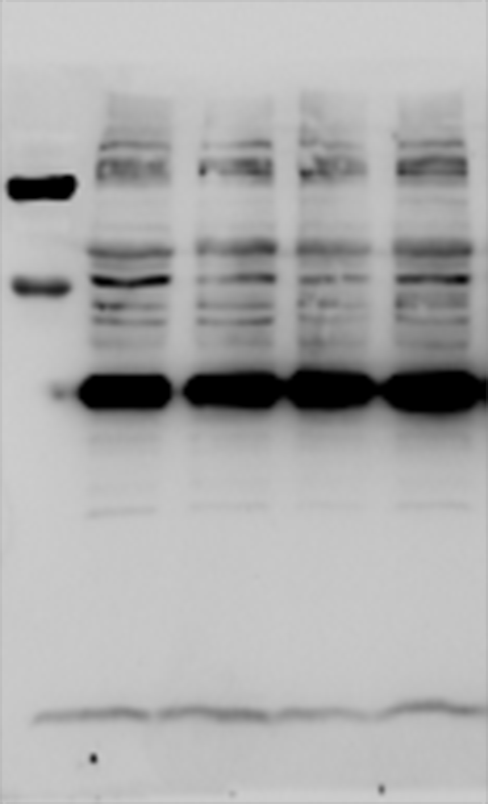

Supplement: Supplementary file 1 [file biology-11-01450-s001.zip › Figure S9.tif]
